# Supplementary figures and images for: Proteomic and genomic characterization of a yeast model for Ogden syndrome
Source: Yeast. 2016 Dec 6;34(1):19–37. doi: 10.1002/yea.3211 (PMC5248646; doi:10.1002/yea.3211)

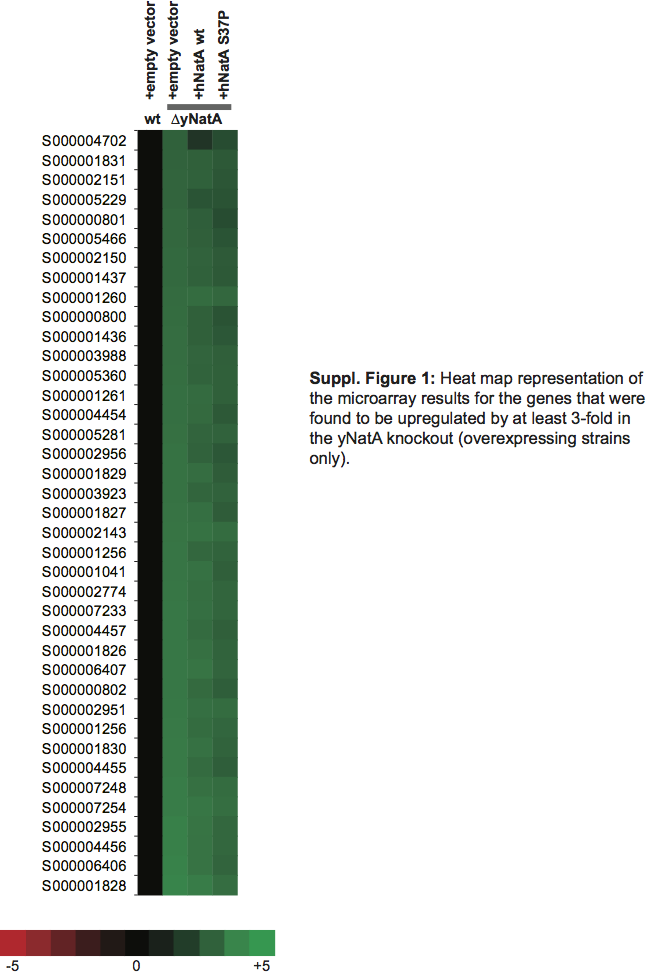

Supplement: Supplementary file 1 — Supporting info item [file YEA-34-19-s001.tiff]
